# Assessment of Patient-Reported Naloxone Acquisition and Carrying With an Automated Text Messaging System After Emergency Department Discharge in Philadelphia

**DOI:** 10.1001/jamanetworkopen.2022.3986

**Published:** 2022-03-24

**Authors:** Anish K. Agarwal, Hareena K. Sangha, Anthony Spadaro, Rachel Gonzales, Jeanmarie Perrone, M. Kit Delgado, Margaret Lowenstein

**Affiliations:** 1 Department of Emergency Medicine, Perelman School of Medicine, University of Pennsylvania, Philadelphia; 2 Leonard Davis Institute of Health Economics, University of Pennsylvania, Philadelphia; 3 Center for Health Care Innovation, University of Pennsylvania, Philadelphia; 4 Division of General Internal Medicine, Department of Medicine, Perelman School of Medicine, University of Pennsylvania, Philadelphia

## Abstract

**Importance:**

A central tenet of harm reduction and prevention of opioid overdose deaths is the distribution and use of naloxone. Patient-centered methods that investigate naloxone acquisition and carrying can guide opioid overdose education and naloxone distribution efforts.

**Objective:**

To assess patients’ self-reported naloxone acquisition and carrying after an emergency department (ED) encounter using automated text messaging.

**Design, Setting, and Participants:**

This cohort study investigated self-reported patient behaviors involving naloxone after ED discharge in a large, urban academic health system in Philadelphia, Pennsylvania. Adult patients who were prescribed or dispensed naloxone and who had a mobile phone number listed in the electronic health record provided informed consent after ED discharge, and data were collected prospectively using text messaging from October 10, 2020, to March 19, 2021. Patients who did not respond to the survey or who opted out were excluded.

**Exposure:**

Automated text message–based survey after ED discharge for patients who were prescribed or dispensed naloxone.

**Main Outcomes and Measures:**

The primary outcome was patient-reported naloxone acquisition, carrying, and use. Descriptive statistics were used to summarize patient demographic characteristics.

**Results:**

Of 205 eligible patients, 41 (20.0%) completed the survey; of those patients, the mean (SD) age was 39.5 (13.7) years, and 21 (51.2%) were women. Fifteen (36.6%) had a personal history of being given naloxone after an overdose. As indicated by the ED record, 27 participants (65.9%) had naloxone dispensed in the ED, and 36 (87.8%) self-reported acquiring naloxone during or after their ED visit. Twenty-four participants (58.5%) were not carrying naloxone in the week before their ED visit. Twenty participants (48.8%) were carrying naloxone after the ED visit, and 27 (65.9%) reported planning to continue carrying naloxone in the future. Of the 24 individuals (58.5%) not carrying naloxone before their ED encounter, 13 (54.2%) reported planning to continue carrying naloxone in the future.

**Conclusions and Relevance:**

In this cohort study of adult patients dispensed or prescribed naloxone from the ED, most reported acquiring naloxone on or after discharge. The ED remains a key point of access to naloxone for individuals at high risk of opioid use and overdose, and text messaging could be a method to engage and motivate patient-reported behaviors in enhancing naloxone acquisition and carrying.

## Key Points

**Question:**

Can text messaging capture patient-reported naloxone acquisition and carrying after an emergency department (ED) encounter?

**Findings:**

In this cohort study of 41 patients discharged from 2 urban EDs, 88% reported acquiring naloxone or a naloxone prescription from the ED. Overall, 66% planned to continue carrying naloxone, including most patients who were not carrying the drug before their ED visits.

**Meaning:**

The findings suggest that text messaging can be used to interact with individuals at high risk for opioid use and overdose, and this automated system offers an opportunity to augment and support ED-based naloxone distribution efforts.

## Teaser

This cohort study uses a text messaging system to survey behavior in patients with naloxone prescriptions after discharge from emergency departments in Philadelphia.

## Introduction

More than 6 of 10 overdose deaths in the US involve an opioid; the number of opioid overdoses has quadrupled in the past 18 years, and an average of 130 US residents die each day because of opioid overdose.^1,2^ As the epidemic worsens, a key strategy for reducing opioid overdose deaths is expanding overdose education and naloxone distribution.^3^ Previous work has found that layperson-directed overdose education and naloxone distribution can safely and effectively empower individuals to administer naloxone to reverse an overdose, and community-based overdose education and naloxone distribution has been associated with decreased mortality.^4^ A recent modeling study of policy strategies for mitigating overdose deaths suggested that wider overdose education and naloxone distribution had the greatest promise for saving lives during the next 5 years.^5^ However, less is known about the optimal ways to engage individuals at high risk for opioid use and overdose and the strategies to increase uptake, carrying, and use of naloxone.

Emergency departments (EDs) offer an opportune touch point and a promising environment to engage individuals at high risk for opioid use and overdose in overdose prevention.^5^ Opioid use disorder (OUD)–related ED visits have increased 100% in the past decade, with an 80% increase in overdose-related ED visits in some areas during the past year.^6^ Naloxone distribution for OUD is becoming common in EDs and is recommended in recent OUD management guidelines.^6,7^ Disparities remain in investigating how patients at risk are identified and prescribed naloxone from the ED, rates of uptake and acquisition among patients (or their friends and family members), and rates of carrying and using naloxone after an ED visit. Although barriers, such as cost or transportation to a pharmacy, introduce complexity in accessing naloxone, understanding the potential reasons for not obtaining and carrying the drug may be partially explained by behavior change models and include cognitive biases, such as availability bias, as well as challenges related to behavior change (eg, low motivation, limited ability, or lack of prompts).^8^ These may be overcome with strategies that target habit formation, including repetition, context cues, and intermittent rewards.^9^

The rapid expansion and adoption of technology has created an emerging and novel method for scalable patient engagement and interaction.^8,10^ Text messaging can be used to quickly capture, analyze, and understand the patient-reported outcomes data and the overall patient journey. Text messaging can also be used to induce behavioral change and collect outcomes in real time.^8,9^ The aim of this study was to deploy a text messaging–based survey after an ED encounter to understand patient behavior and perceptions toward acquisition, carrying, and use of naloxone. The hypothesis was that discharged ED patients prescribed naloxone would report low rates of acquisition and carrying.

## Methods

### Study Population, Setting, and Time Period

This cohort study was designed to develop a system-level method of engaging patients to understand patient-reported outcomes, including naloxone acquisition, carry, and use rates, before or after an ED encounter. We invited all patients discharged with a traditional naloxone prescription (paper or electronic) from 2 academic urban EDs at the Hospital of the University of Pennsylvania and Penn Presbyterian Medical Center to participate in this study between October 10, 2020, and March 19, 2021. The decision to prescribe naloxone was at the prescriber’s discretion on discharge. Emergency departments had different dispensing capacities and protocols, and thus, variation in practice was observed. All patients received a prescription for naloxone to either be filled at a pharmacy or dispensed to the patient on ED discharge, which was documented in the electronic health record (EHR). Other eligibility criteria included that the patient be aged 18 years or older, have a mobile phone number listed in the EHR, and be discharged from either ED. Data on race and ethnicity were collected but not reported given the small sample size to protect participants’ privacy. The institutional review board of the University of Pennsylvania approved this study, and patients provided electronic informed consent. This study followed the Strengthening the Reporting of Observational Studies in Epidemiology (STROBE) reporting guideline.^11^

### Recruitment

Eligible patients were identified from automated daily EHR-based ED reports of discharged patients. The phone number of each eligible patient was entered into an automated text messaging platform (Way to Health, University of Pennsylvania), which was programmed to prospectively collect data directly from patients on their perspectives about care (eAppendix in the Supplement).^12^ The initial text message obtained electronic consent to receive subsequent text messages, provided study information, and collected self-reported patient responses. In addition, the text message provided directions for patients to opt out at any time. No patient incentives were included in this study.

### Data Collection

Patients were sent the first text message 24 hours after ED discharge or the next business day after their ED visit. After consenting to participate, the patients were asked to report the following: (1) whether they had acquired naloxone after their ED visit, (2) if they carried naloxone during the 7 days before their visit, (3) if they had ever reversed an overdose in someone else or had themselves experienced an overdose reversal, and (4) if they planned to continue to carry naloxone in the future. The survey questions were developed by the research team and designed to be easily formatted to a mobile phone. The questions were pilot tested for functionality by 5 patients, who were not included in the analysis, and no changes were made. A member of the research team (H.K.S.) who was not involved with clinical care performed the enrollment and follow-up. Patient information was deidentified and stored securely. A member of the research team trained in medical record abstraction (H.K.S.) obtained demographic information from the EHR.

### Statistical Analysis

The primary outcome of interest was patient-reported acquisition and carrying of naloxone after an ED visit. Secondary outcomes included use of naloxone to reverse an overdose in another person or if the patient experienced an overdose and reversal themselves, and rates of carrying naloxone. Descriptive statistics are reported as numbers and percentages and are used to summarize patient demographic characteristics. Comparisons were done with χ^2^ tests, which were used for categorical variables across respondents, and 2-sided *t* tests, which were used for continuous variables across respondents and nonrespondents, with Stata, version 16.1 (StataCorp, LLC). A 2-sided α = .025 was considered to be statistically significant.

## Results

A total of 205 eligible patients were either discharged with a naloxone prescription or dispensed naloxone while in the ED. All were sent a postdischarge text message inviting them to participate in the study, and 41 (20.0%) patients provided electronic consent and completed all questions (eAppendix in the Supplement). These patients had a mean (SD) age of 39.5 (13.7) years; 21 (51.2%) were women and 20 (48.8%) men. Seventeen (41.5%) had stable permanent housing, and 31 (75.6%) had Medicaid or Medicare. No participants opted out after providing consent. Of the 164 patients who did not participate, 1 individual opted out, and the remaining 163 provided no response. The text messaging platform was unable to assess out-of-service phone numbers. Participants who did not respond were excluded. Participants were demographically similar to nonrespondents in terms of age, sex, and insurance status, but there were significant differences in educational level, with respondents reporting their educational level more than nonrespondents (23 of 41 [56.1%] vs 10 of 164 [6.1%], *P* < .001), and housing status, with respondents reporting their housing status more than nonrespondents (25 of 41 [61.0%] vs 37 of 164 [22.6%], *P* < .001) (Table 1).

**Table 1.** Participant Demographic Characteristics and Descriptive Statistics

Characteristic	Respondents, No. (%) (n = 41)	Nonrespondents, No. (%) (n = 164)	*P* value	Total eligible, No. (%) (n = 205)
Age, mean (SD), y	39.5 (13.7)	42.6 (16.3)	.26	42 (15.8)
Sex				
Men	20 (48.8)	100 (61.0)	.22	120 (58.5)
Women	21 (51.2)	64 (39.0)		85 (41.5)
Educational level				
Some high school or less	2 (4.9)	1 (0.6)	<.001	3 (1.5)
High school diploma or GED	8 (19.5)	3 (1.8)		11 (5.4)
Some college or college degree	13 (31.7)	5 (3.0)		18 (8.8)
Graduate or professional degree	0	1 (0.6)		1 (0.5)
Unknown or not reported	18 (43.9)	154 (93.9)		172 (83.9)
Housing status				
Permanent housing			<.001	
Stable	17 (41.5)	17 (10.4)		34 (16.6)
Unstable	2 (4.9)	5 (3.0)		7 (3.4)
Unhoused, recovery house, or shelter	6 (14.6)	15 (9.1)		21 (10.2)
Unknown or not reported	16 (39.0)	127 (77.4)		143 (69.8)
Insurance status				
Medicaid	27 (65.9)	105 (64.0)	.28	132 (64.4)
Medicare	4 (9.8)	27 (16.5)		31 (15.1)
Private/employer	7 (17.1)	15 (9.1)		22 (10.7)
Uninsured	1 (2.4)	13 (7.9)		14 (6.8)
Unknown or not reported	2 (4.9)	4 (2.4)		6 (2.9)

Abbreviation: GED, General Educational Development.

Twenty-seven participants (65.9%) were dispensed naloxone in the ED, 15 (36.6%) had a personal history of being given naloxone after an overdose, and 11 (26.8%) reported administering naloxone to another person to reverse an overdose in the past. Most participants (36 [87.8%]) reported being prescribed or dispensed naloxone to take home after their ED encounter. Twenty-four participants (58.5%) had not previously carried naloxone in the 7 days before their ED visit. Of those individuals who reported carrying naloxone before their ED encounter, participants carried naloxone a mean (SD) of 4.7 (2.8) days per week. Twenty participants (48.8%) were actively carrying naloxone when surveyed after their ED visit, and 27 (65.9%) reported planning to continue carrying naloxone in the future. Of the 24 individuals (58.5%) who did not carry naloxone before the ED visit, 8 (33.3%) were actively carrying when surveyed after discharge, and 13 (54.2%) planned to continue carrying naloxone in the future. Compared with individuals given a prescription for naloxone, individuals dispensed naloxone in the ED more often reported receiving it (25 of 27 [92.6%] vs 11 of 14 [78.6%]), actively carrying it (17 of 27 [63.0%] vs 3 of 14 [21.4%]), and planning to continue carrying it (20 of 27 [74.1%] vs 7 of 14 [50.0%]) (Table 2).

**Table 2.** Participant Text Messaging Responses

Survey question	No. (%)			
	Naloxone dispensed in ED (n = 27)		Prescription for naloxone provided (n = 14)	
	Yes	No	Yes	No
“Were you given naloxone (or Narcan) to take home with you after your recent visit?”	25 (92.6)	2 (7.4)	11 (78.6)	3 (21.4)
“Have you ever carried Narcan before your recent visit?”	14 (51.9)	13 (48.1)	3 (21.4)	11 (78.6)
“Thinking back to the week before your visit, how many days did you carry Narcan?”^a^	4.7 (2.8)	NA	4.5 (2.7)	NA
“Have you ever used Narcan on another person to reverse overdose?”	8 (29.6)	19 (70.4)	3 (21.4)	11 (78.6)
“Has anyone ever given you Narcan to reverse an overdose?”	11 (40.7)	16 (59.3)	4 (28.6)	10 (71.4)
“Are you carrying Narcan now?”	17 (63.0)	10 (37.0)	3 (21.4)	11 (78.6)
“Do you plan to continue carrying Narcan?”	20 (74.1)	7 (25.9)	7 (50.0)	7 (50.0)

Abbreviations: ED, emergency department; NA, not applicable.

^a^ Results for this question are reported as mean (SD).

## Discussion

Opioid overdose remains a persistent and devastating threat. Reversing overdose using naloxone is effective, yet barriers persist in making naloxone available and accessible and promoting its use and rescue. The benefits of overdose education and naloxone distribution are well described, but less is known about methods of engaging individuals at high risk for opioid use and overdose at key touch points within the health system. In this study, we used a novel scalable, patient-centered method of engaging patients after an ED visit for OUD to enhance naloxone acquisition, carrying, and use. As health systems strive to improve the outcomes of individuals with OUD, it is critical to place an intentional focus on naloxone distribution and carrying. Emergency departments provide care for individuals with overdose and OUD and thus are ideal for this investigation. The study had 3 key findings. First, using a simple and unincentivized (eg, no financial or other incentive) text messaging survey, we engaged 20.0% of a population that is historically difficult to reach after an ED encounter to learn about their experiences accessing, using, and carrying naloxone.^13,14^ Second, most patients did not carry naloxone before their ED visits despite more than a third of patients reporting having an overdose reversed in the past and more than a quarter reporting using naloxone to reverse an overdose on another person in the past. Third, ED visits represent an opportunity to improve naloxone carrying as roughly half of the patients who responded were carrying after their ED visit, two-thirds planned to continue carrying, and more than half of those who did not carry before planned to continue carrying afterward.

Sustained behavior change is challenging, and the stigma associated with opioid use and OUD provides additional and complex nuances in addressing care. Naloxone distribution has increased with efforts to improve access in the community and nontraditional settings (eg, public transportation or libraries), but there are still substantial missed opportunities to provide naloxone for patients at risk of overdose in EDs and other health care settings.^7^ Emergency department visits provide high-impact touch points for patients with OUD, as they may be seeking OUD-related care and may be mentally primed to consider carrying naloxone. In this study, we found that of the patients not carrying naloxone before the ED visit, 54.2% reported a plan to continue carrying in the future. A key component of motivating behavioral change is creating spaces for patients to provide input and feedback on their perspectives and experiences using naloxone. Often, the physical limitations attributable to increasing ED case volumes and crowding create an environment less conducive to open discussions between clinicians and individuals with OUD, but today’s digital era may provide a space to allow patients to voice their opinions.^15,16,17^

Of note, this study did not include additional education or communication with the patient on the importance of naloxone but rather engaged them via text messaging to ask them short, simple questions. Future work will need to investigate scalable motivational strategies to sustain long-term naloxone carrying after an ED encounter. In alignment with past research, our study reinforced that ED-based naloxone dispensing mechanisms are critical to obtaining and carrying naloxone compared with prescriptions, which then need to be filled and could introduce additional barriers to access.^7,18^ These early insights may help quality improvement and operational and administrative efforts to support OUD and addiction care. We showed the feasibility of using text messaging, with a response rate (20.0%) similar to or higher than other published ED patient satisfaction surveys.^19,20^ Important next steps in our research include investigating methods to integrate motivational messaging and reminders to improve and sustain naloxone carrying. To our knowledge, this study is among the first to use these methods from the ED and supports the growing body of literature toward patient-centered learning health system design.

### Strengths and Limitations

This study has strengths. It applied a novel, low-tech method to engage patients and collect patient-reported information to inform health care in a meaningful way. It did not burden clinical staff with identifying, obtaining consent, or collecting data from patients. This approach was patient centered and provided health systems with a method of expanding the core concepts of learning health systems and continuous quality improvement.

The study also has limitations. First, it was conducted within a single academic health system across 2 urban EDs. Although incorporating data collection via text messaging into routine care allowed us to solicit responses from all patients, selection and nonresponder bias remain present, in that individuals must opt in to the text messaging survey. In addition, as we recruited only individuals with a mobile phone number, these findings are not generalizable to those without stable access to a mobile phone. Response rates were not high but were hard to compare because, to our knowledge, surveys delivered using text messaging for individuals at high risk for OUD are limited. Furthermore, participants needed to have regular access to a text message–capable device. Future efforts to develop methods of engaging patients to understand their experiences carrying naloxone specific to education and housing status would benefit from assessing heterogeneity in patient responses after discharge, accounting for the degree of nonresponse. Nonetheless, to our knowledge, this study is among the first to prospectively engage and begin to investigate patient-reported carrying and perceptions of naloxone in a remote and automated fashion immediately after an ED visit. In addition, we relied on self-report, so although participants were notified that data collection was only for research and would not affect their clinical care, responses may have been subject to social desirability bias.

## Conclusions

This cohort study used a novel approach that suggests an early and important disparity in naloxone carrying before and after an ED encounter and plan to continue carrying. It begins to chart the important path forward of scalable patient-centered efforts to motivate, augment, and sustain behavior change in naloxone carrying.

## Notes

Supplement. **eAppendix.** Text Messaging Script
