# Supplementary material for: Assessment of Patient-Reported Naloxone Acquisition and Carrying With an Automated Text Messaging System After Emergency Department Discharge in Philadelphia
Source: JAMA Netw Open. 2022 Mar 24;5(3):e223986. doi: 10.1001/jamanetworkopen.2022.3986 (PMC8948533; doi:10.1001/jamanetworkopen.2022.3986)
Supplement: Supplement. — eAppendix. Text Messaging Script [file jamanetwopen-e223986-s001.pdf]

Supplementary Online Content

Agarwal AK, Sangha HK, Spadaro A, et al. Assessment of patient-reported naloxone acquisition and carrying with an automated text messaging system after emergency department discharge in Philadelphia. *JAMA Netw Open*. 2022;5(3):e223986. doi:10.1001/jamanetworkopen.2022.3986

eAppendix. Text Messaging Script

This supplementary material has been provided by the authors to give readers additional information about their work.

© 2022 Agarwal AK et al. *JAMA Network Open*.

eAppendix. Text Messaging Script

Consent: **Q1**

“Hi, this is Penn Medicine checking in about your recent visit. Text ‘YES’ if we can ask a few questions about medication your received during your visit. Text ‘STOP’ anytime to opt-out.

Texting is not secure. Other people may be able to see information in text messages. This information helps guide future patient care and will not be passed along to your care team or impact your treatment. By texting back ‘YES’ you are accepting this risk. Message & data rates may apply."

Q2:
“Were you given naloxone (or “Narcan”) to take home with you after your recent visit? Please text back Yes or No.”

LOGIC:

- If response = Y, N, yes, or no → Proceed to **Q3**
- If response ≠ Y, N, yes or no → REPLY *"I’m sorry. We don’t understand. Please text back Y or N.”*

Q3:
“Have you ever carried Narcan before your recent visit? Please text back Yes or No.”

LOGIC:

- If response = Y, yes → Proceed to **Q4**
- If respond = N, no → Proceed to **Q5**
- If response ≠ Y, N, yes or no → REPLY *"I’m sorry. We don’t understand. Please text back Y or N.”*

Q4:
“Thinking back to the week before your visit, how many days did you carry Narcan? Please text back a number 1-7.”

LOGIC:

- If response = numeric value 1-7 or one, two, three, four, five, six, seven → Proceed to **Q5**
- If response ≠ 1-7 or one-seven → REPLY *"I’m sorry. We don’t understand. Please text back a number 1-7.”*

Q5:
“Have you ever used Narcan on another person to reverse overdose? Please text back Yes or No.”

LOGIC:

- If response = Y, N, yes, or no → Proceed to **Q6**
- If response ≠ Y, N, yes or no → REPLY *"I’m sorry. We don’t understand. Please text back Y or N.”*

Q6:
“Has anyone ever given you Narcan to reverse an overdose? Please text back Yes or No.”

LOGIC:

© 2022 Agarwal AK et al. *JAMA Network Open*.

- If response = Y, N, yes, or no → Proceed to **Q7**
- If response ≠ Y, N, yes or no → REPLY *"I'm sorry. We don't understand. Please text back Y or N."*

Q7:
"Are you carrying Narcan now? Please text back Yes or No."

LOGIC:

- If response = Y, N, yes, or no → Proceed to question **8**
- If response ≠ Y, N, yes or no → REPLY *"I'm sorry. We don't understand. Please text back Y or N."*

Q8:
"Do you plan to continue carrying Narcan? Please text back Yes or No."

LOGIC:

- If response = Y, N, yes, or no → **END**
- If response ≠ Y, N, yes or no → REPLY *"I'm sorry. We don't understand. Please text back Y or N."*

© 2022 Agarwal AK et al. *JAMA Network Open*.
